# Structural Monitoring of Metro Infrastructure during Shield Tunneling Construction

**DOI:** 10.1155/2014/784690

**Published:** 2014-06-15

**Authors:** L. Ran, X. W. Ye, G. Ming, X. B. Dong

**Affiliations:** ^1^Hangzhou Metro Group Co., Ltd., Hangzhou 310020, China; ^2^Department of Civil Engineering, Zhejiang University, Hangzhou 310058, China

## Abstract

Shield tunneling construction of metro infrastructure will continuously disturb the soils. The ground surface will be subjected to uplift or subsidence due to the deep excavation and the extrusion and consolidation of the soils. Implementation of the simultaneous monitoring with the shield tunnel construction will provide an effective reference in controlling the shield driving, while how to design and implement a safe, economic, and effective structural monitoring system for metro infrastructure is of great importance and necessity. This paper presents the general architecture of the shield construction of metro tunnels as well as the procedure of the artificial ground freezing construction of the metro-tunnel cross-passages. The design principles for metro infrastructure monitoring of the shield tunnel intervals in the Hangzhou Metro Line 1 are introduced. The detailed monitoring items and the specified alarming indices for construction monitoring of the shield tunneling are addressed, and the measured settlement variations at different monitoring locations are also presented.

## 1. Introduction

Establishment of the urban metro system will not only efficiently alleviate the problem of urban traffic congestion but also vigorously promote the integrated development among different urban regions [1–3]. Deep excavation and shield tunneling are two critical issues during construction of the metro infrastructure such as metro stations, metro tunnels, and metro-tunnel cross-passages. A significant matter with great concern lies in ensuring the safety of the metro infrastructure at the construction stage, especially when the in-construction metro lines are adjacent to other metro infrastructures or beneath key engineering structures. In the last two decades, a considerable number of civil engineering structures have been instrumented with online structural health monitoring (SHM) systems [4–10]. Investigations pertaining to structural damage detection and safety condition assessment of the concerned structures by use of the long-term measurement data have been conducted by massive researchers worldwide [11–15], while less percent of them have been focused on the underground structures.

In recent years, a significant amount of research has been devoted to the technological progress of the shield tunneling construction and the relevant uncertainty analysis. Goel [16] presented the current status of tunneling and its future potential in India with emphasis on tunneling projects for hydropower developments as well as the tunneling technologies for planning, design, and construction. Boubou et al. [17] analyzed the tunneling-induced ground movement and its correlation with the operation parameters of the tunnel boring machine (TBM) by use of a nonlinear least square approximation and an artificial neural network (ANN). Špačková and Straub [18] developed a dynamic Bayesian network (DBN) for probabilistic assessment of tunnel construction performance to facilitate the quantification of uncertainties in the construction process and of the risk from extraordinary events. Fernandez and Moon [19, 20] conducted a theoretical investigation on the significance of the hydromechanical interaction, and the effectiveness of the proposed analytical solution was verified through numerical analysis. Liao et al. [21] studied three typical cases of shield crossings based on the successful engineering projects in Shanghai soft ground and presented the risks of shield crossing sensitive buildings and subways, ground movement prediction and control regulations, and the settings of shield driving parameters.

Regarding the safety monitoring and condition assessment of the underground structures, Bhalla et al. [22] addressed the major technological issues and challenges associated with the structural monitoring of underground structures and presented a detailed review of the available sensor technologies and methods for comprehensive monitoring with special emphasis on conditions encountered underground. Sharma et al. [23] investigated the effect of a large excavation on the deformation of two adjacent mass rapid transit (MRT) tunnels using field monitoring data and finite element analysis. Klar and Linker [24] presented a feasibility study of automated detection of the smuggling tunnels with the development of the smart underground security fence using Brillouin optical time domain reflectometry (BOTDR). Shao and Macari [25] systematically introduced an effective and reliable method in predicting the deformation for the deep excavation, relying on the accurate field-measured displacement as the input information and feedback to a numerical model of the deep excavation. Kavvadas [26] described the types of ground deformation measurements often used in tunneling, the difficulties in obtaining ground deformation measurements, the methods commonly used for evaluation, and the application of these measurements in establishing early warning systems. Bennett et al. [27] introduced the challenges involved with a wireless sensor network (WSN) installation in an underground environment and described two case studies of field trials of WSN systems.

## 2. Description of Metro-Tunnel Shield Sections

### 2.1. Scope of Shield Tunnel Intervals

**Figure 1:**
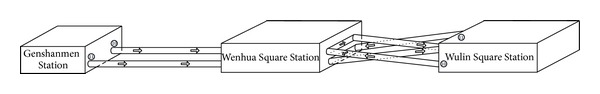
Layout of tunneling construction of metro shield intervals.

As illustrated in Figure 1, the studied shield tunnel intervals, namely, Genshanmen Station-Wenhua Square Station interval and Wenhua Square Station-Wulin Square Station interval, are two important shield tunneling construction sections in Hangzhou Metro Line 1, which is the first metro line of the urban rapid rail transit system in Hangzhou, China. For the Genshanmen Station-Wenhua Square Station interval, the shield tunnel extends from the Wenhua Square Station and arrives at Wenhui Road after turning right from the Zhongshan North Road. This shield tunnel line will travel beneath 13 residential buildings of Zhaohui community as well as the bridge piers of the Shangtang Viaduct, and part of the shield tunnel also will pass beneath two rivers. As per the Wenhua Square Station-Wulin Square Station interval, the shield tunnel starts from the east of the Wulin Square Station and travels beneath Huancheng North Road and the Beijing-Hangzhou Grand Canal. It gradually enters Zhongshan North Road from the West Lake Wenhua Square and then reaches the Wenhua Square Station being as two overlapping tunnels.

### 2.2. Geological Conditions

The geological structure of the shield tunnel section consists of a considerable amount of silty soils and sandy soils. Due to the differences of accumulation periods and consolidation conditions, the compaction extent varies from loose condition to medium density in the vertical direction. The properties of the soil layers are different, and the general thickness of the soil layers is about 20 m. The silt soft soils and clayey soils are buried beneath with a dense condition and the thickness of the soil layers is about 40~45 m. The depth of the bottom bedrock is about 55~63 m from the ground surface. According to the geological properties and forming conditions, the foundation soils are divided into different soil layers, as listed in Table 1 in detail. The shield tunnels of the Genshanmen Station-Wenhua Square Station interval mainly pass through the soil layers *④*_4_, *⑥*_1_, *⑥*_2_, *⑦*_1_, *⑧*_2_, and *⑨*_1_, while the shield tunnels of the Wenhua Square Station-Wulin Square Station interval go through the soil layers *④*_2_, *④*_3_, *⑥*_1_, *⑦*_1_, and *⑦*_2_.

## 3. Organization of Shield Tunnel Construction

### 3.1. General Planning of Shield Tunneling

After a comprehensive investigation on the type and characteristics of the metro-tunnel shield sections, various construction teams are established, namely, shield construction team, tunnel segment construction team, and integration construction team. During the whole construction process, the shield construction should be prepared in advance and the accessory structures will be constructed during the construction intermittence of the main structures. The construction time nodes are settled in accordance with the completion of the construction of the milestone part of the project. The timely completion of the construction of the shield tunnel intervals is ensured through harmonious construction planning. By doing so, the general organization of the shield tunnel construction will be legitimately controlled.

**Figure 2:**
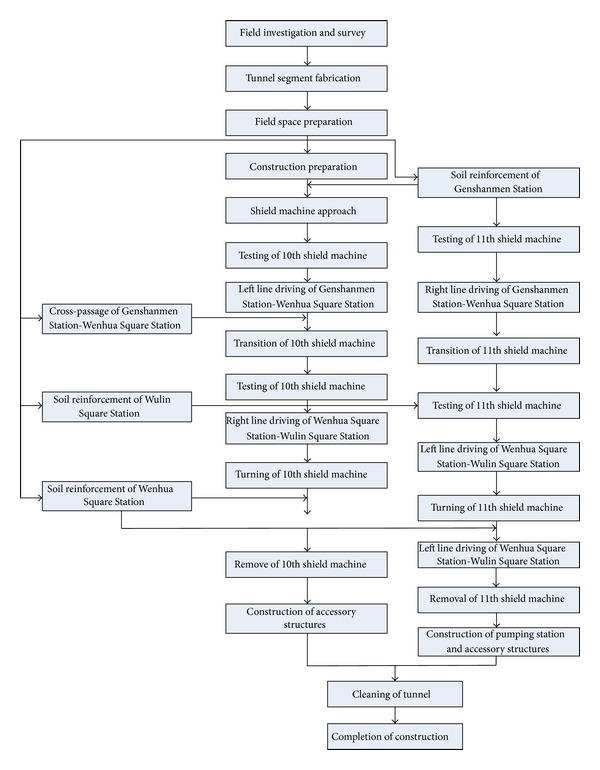
Framework of shield tunnel construction.

**Figure 3:**
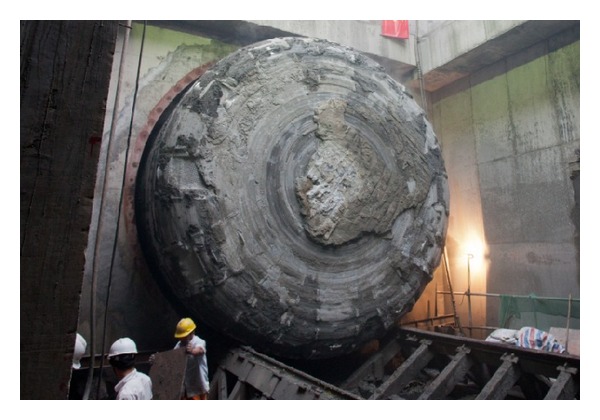
Photo of field shield tunnel construction.

As illustrated in Figure 2, two shield tunneling machines are used in the concerned shield tunnel intervals. The 10th shield machine starts from the left line at the Genshanmen Station and will be removed from the Wenhua Square Station. It will be moved again to the right line of the Wulin Square Station and reaches the Wenhua Square Station and then ends at Wulin Square Station. The 11th shield machine starts from the right line at the Genshanmen Station and is removed from the Wenhua Square Station. It will be moved again to the left line of the Wulin Square Station and arrives at the Wenhua Square Station and then ends at Wulin Square Station.  Figure 3 shows the site photo of the shield tunnel construction.

### 3.2. Freezing Construction of Cross-Passages

**Figure 4:**
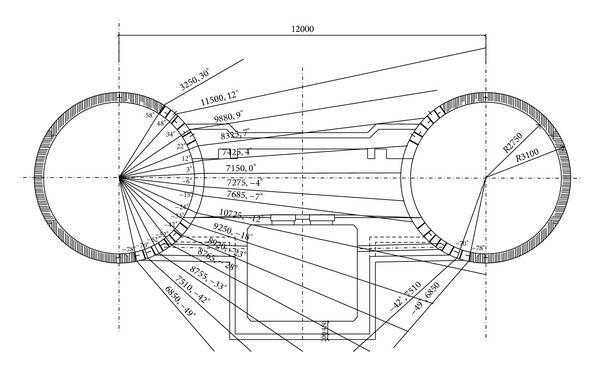
Arrangement of freezing tubes.

**Figure 5:**
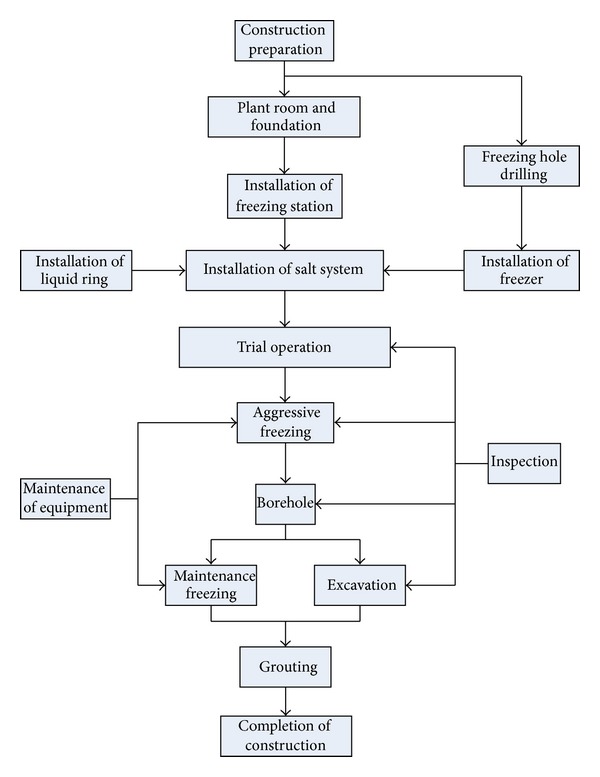
Procedure of artificial ground freezing construction.

Following the Chinese code of metro design, a cross-passage between two tunnels should be set up if the continuous length of one of the tunnels is larger than 600 m. On the basis of the geological conditions of the shield tunnel sections, the soils around the tunnels are reinforced through the horizontal freezing and then the tunnel linings are constructed after excavation. By doing so, a high-strength sealing frozen curtain will be formed to guarantee the safety of excavation construction.  Figure 4 shows the detailed deployment of the freezing tubes during the artificial ground freezing construction. The procedure of the artificial ground freezing construction is illustrated in Figure 5.

## 4. In-Construction Monitoring and Safety Evaluation

### 4.1. Design Principles for Metro Infrastructure Monitoring

**Figure 6:**
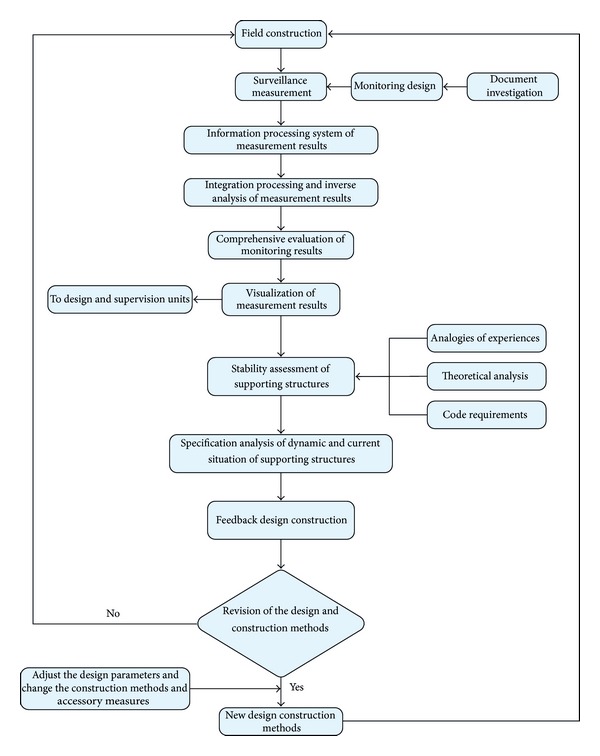
Flowchart of systematical surveillance and measurement.

For the managers of metro infrastructure, the implementation of efficient surveillance and measurement of engineering projects with large risks is crucial such as the deep excavations with a depth over 5 m and the shield tunnel intervals. The execution of the informationalised construction through in-construction monitoring can make the engineering measurement normal and standard. The design principles for monitoring the metro infrastructure include the following: (i) all the monitoring items are effectively synthesized and the measurement data can be interactively verified; (ii) a comprehensive monitoring scope should be established to ensure that the measurement data are promptly acquired with a continuous and accurate manner; (iii) the mature and advanced measurement equipment is adopted and should be protected during installation; (iv) the critical parameters in the structural design are monitored for optimization; (v) special attention should be paid to the key construction sections for dense monitoring; (vi) the monitoring types, locations, and protection measures are determined based on the realistic construction; and (vii) the monitoring method is selected in accordance with the rules of concision and cost-effectiveness.  Figure 6 illustrates the flowchart of the systematical surveillance and measurement during metro-tunnel shield construction.

### 4.2. Monitoring Items and Result Analysis

**Figure 7:**
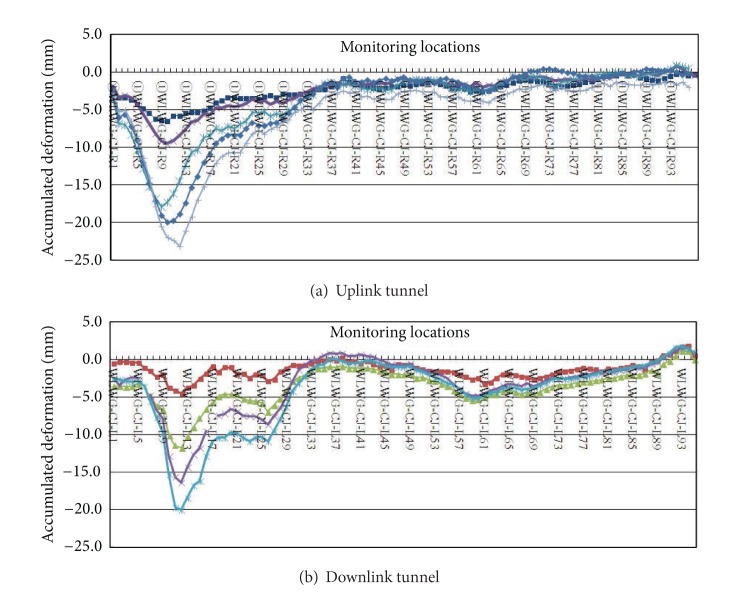
Monitored settlement variations at different monitoring locations.

In this project, the monitoring items include the settlement, inclination and cracking of the surrounding buildings, the vertical displacement of the adjacent underground colligation pipelines, the settlement of the ground surface, the underground water level, the sink deformation of the tunnel vault, the tunnel bottom heave, and the tunnel clearance convergence. The alarming indices are generally stipulated based on the total variations and the variation velocities of the monitoring parameters, and the warning value of the total variation should not be larger than the design value.  Table 2 lists the specified alarming indices for monitoring of the shield tunnel construction.  Figure 7 shows the measured settlement variations at different monitoring locations of the uplink and downlink tunnels at the Wenhua Square Station-Wulin Square Station interval.

## 5. Conclusions

Development and utilization of the urban underground space have gained increasing concerns from the urban administrative agency and the civil engineering community, which has been a significant flag for urban modernization. In the past years, the design and construction of the urban underground structures are considered in terms of the strength and stability of the structural components. However, the deformation control design is deemed to be dominant for the deep excavation of the metro infrastructure due to the increasing number of the surrounding buildings as well as the scope and depth of the metro-tunnel shield construction. In this connection, monitoring of the metro infrastructure during the shield tunnel construction becomes important which will facilitate the condition tracking and safety evaluation of the metro infrastructure with the help of the long-term measurement data.

## Figures and Tables

**Table 1:** Property description of soil layers in shield tunnel section.

Number	Soil layer	Detail description
*①*_1_	Miscellaneous fill soil	Mainly composed by construction and domestic waste (0.4~3.0 m)
*①*_2_	Plain fill soil	Generally composed by the silty soil with organic matters (0.4~3.4 m)
*①*_3_	Silty soil	Containing lots of humic substances and scrap micas (0.6 m)
*②*_1_	Silty clay	With much iron oxides and scrap micas (0.6~1.8 m)
*②*_2_	Clayey silt	With much iron oxides and scrap micas (0.3~2.2 m)
*②*_3_	Sandy silt	With much iron oxides and scrap micas
*③*_1_	Clayey silt	With organic matters and scrap micas
*③*_2_	Clayey silt	With organic matters and scrap micas
*③*_3_	Sandy silt	With organic matters and scrap micas
*③*_4_	Clayey silt	With organic matters and scrap micas
*③*_5_	Sandy silt	With organic matters and scrap micas
*③*_6_	Sandy silt	With organic matters, iron oxides, and scrap micas
*③*_7_	Silty clay	With organic matters and scrap micas
*④*_1_	Silty soft clay	With organic matters and silty soils (2.3~6.8 m)
*④*_2_	Silty soil	With organic matters and sludge soils (2.3~7.0 m)
*④*_3_	Silty soil	With organic matters (3.4~7.9 m)
*④*_4_	Clayey silt	With organic matters, scrap micas, and silty clay
*⑥*_1_	Silty soil	With organic matters (3.7~9.4 m)
*⑥*_2_	Silty soil	With organic matters (2.1~6.6 m)
*⑦*_1_	Clay	With organic matters and silty soils (2.3~6.9 m)
*⑦*_2_	Clay	With organic matters and silty soils
*⑧*_2_	Gray clay	With organic matters and silts (2.0~10.4 m)
*⑨*_1_	Silty clay	With organic matters and scrap micas (1.6~5.2 m)
*⑨*_2_	Silty clay	With organic matters and scrap micas (0.6~4.8 m)

**Table 2:** Alarming indices for construction monitoring of shield tunneling.

Item	Alarming index		
	Daily variation	Design value	Warning value
Deformation of adjacent underground colligation pipelines	2 mm	20 mm	80% of design value
Settlement of adjacent ground surface	3 mm	+10 mm/−30 mm	
Vertical displacement of surrounding buildings	2 mm	20 mm	
Settlement of tunnel	2 mm	20 mm	
Tunnel clearance convergence	2 mm	30 mm	
